# Discovery of long‐distance gamete dispersal in a lichen‐forming ascomycete

**DOI:** 10.1111/nph.14714

**Published:** 2017-08-07

**Authors:** Cecilia Ronnås, Silke Werth, Otso Ovaskainen, Gergely Várkonyi, Christoph Scheidegger, Tord Snäll

**Affiliations:** ^1^ Swedish Species Information Centre Swedish University of Agricultural Sciences Box 7007 Uppsala S‐75007 Sweden; ^2^ Institute of Plant Sciences University of Graz Holteigasse 6 Graz 8010 Austria; ^3^ Department of Biosciences University of Helsinki PO Box 65 Helsinki FI‐00014 Finland; ^4^ Centre for Biodiversity Dynamics Department of Biology Norwegian University of Science and Technology Trondheim N‐7491 Norway; ^5^ Friendship Park Research Centre Finnish Environment Institute SYKE Lentiirantie 342B Kuhmo FI‐88900 Finland; ^6^ Swiss Federal Institute for Forest Snow and Landscape Research WSL Zürcherstr. 111 Birmensdorf CH‐8903 Switzerland

**Keywords:** asexual, clonal, dispersal, gamete, lichen, long distance, sexual, short distance

## Abstract

- Accurate estimates of gamete and offspring dispersal range are required for the understanding and prediction of spatial population dynamics and species persistence. Little is known about gamete dispersal in fungi, especially in lichen‐forming ascomycetes. Here, we estimate the dispersal functions of clonal propagules, gametes and ascospores of the epiphytic lichen *Lobaria pulmonaria*.
- We use hierarchical Bayesian parentage analysis, which integrates genetic and ecological information from multiannual colonization and dispersal source data collected in a large, old‐growth forest landscape.
- The effective dispersal range of gametes is several hundred metres to kilometres from potential paternal individuals. By contrast, clonal propagules disperse only tens of metres, and ascospores disperse over several thousand metres.
- Our study reveals the dispersal distances of individual reproductive units; clonal propagules, gametes and ascospores, which is of great importance for a thorough understanding of the spatial dynamics of ascomycetes. Sexual reproduction occurs between distant individuals. However, whereas gametes and ascospores disperse over long distances, the overall rate of colonization of trees is low. Hence, establishment is the limiting factor for the colonization of new host trees by the lichen in old‐growth landscapes.

## Introduction

Dispersal is a process of fundamental importance for the spatial dynamics, evolution and migration of populations (Bullock *et al*., 2002; Kokko & López‐Sepulcre, 2006). For many species, habitats are naturally or anthropogenically fragmented, and populations of species with poor dispersal ability may become isolated. Because isolation decreases the chance of re‐colonization in habitat patches after local extinction (Hanski, 1999), species with poor dispersal ability may go extinct from entire landscapes (Hanski, 1999; Thomas, 2000).

Dispersal between habitat patches requires a dispersal mode which enables the crossing of unsuitable habitats. In sessile organisms, dispersal is restricted to the reproductive phase. Many of these species can reproduce both clonally and sexually. Some ascomycete moulds (including *Aspergillus* and *Cladosporium*) can be dispersed over hundreds of kilometres with massive amounts of conidia (small clonal propagules) suited for wind dispersal (Lacey, 1996). They are also amongst the most abundant fungal diaspores in the air (Lacey, 1996). In general, however, clonal reproduction produces fairly large diaspores which enhances short‐distance dispersal, whereas sexual reproduction produces small diaspores enhancing long‐distance dispersal (Tackenberg, 2003; Scheidegger & Werth, 2009; Werth *et al*., 2014). These two dispersal modes with different propagule sizes and resulting dispersal distances have large effects on local population growth, pattern and rate of spatial spread, metapopulation dynamics and genetic diversity (Snyder & Chesson, 2003; Soons & Ozinga, 2005; Zhang & Zhang, 2007; Marco *et al*., 2011; Johansson *et al*., 2012). However, the success of sexual dispersal also depends on the availability of breeding partners, either by direct contact between neighbouring individuals or thanks to the dispersal of mobile gametes.

The dispersal range of propagules and gametes is still poorly known in most ascomycetes. These fungi have evolved different modalities of sexual reproduction (Debuchy *et al*., 2010; Taschen *et al*., 2016). One modality is similar to pollination in vascular plants – by mating of a female cell with male gametes (Bistis, 1981; Bultman & White, 1988; Kohn, 1993; Healy *et al*., 2013). Although the process of mating is well known in several non‐lichenized fungi (Bistis, 1981; Coppin *et al*., 2005; Fleissner *et al*., 2005, 2009; Taschen *et al*., 2016), knowledge of the dispersal of gametes remains rudimentary in ascomycetes, including lichenized species.

Lichen‐forming ascomycetes provide the valuable opportunity to increase our understanding of the dispersal of propagules and gametes in fungi. The epiphytic lichen *Lobaria pulmonaria* represents an excellent and well‐investigated model system (Scheidegger & Werth, 2009; Belinchón *et al*., 2017). Lichen‐forming ascomycetes form a perennial, complex symbiotic association of fungi (mycobiont) and photosynthetic green algae and/or cyanobacteria (photobionts). Lichen‐forming ascomycetes disperse with sexually produced ascospores and/or by clonal propagules. In many cases, clonal propagules of lichen‐forming ascomycetes are relatively large symbiotic structures which facilitate co‐dispersal of the symbionts (Dal Grande *et al*., 2012), such as soredia, isidia and thallus fragments, which do not reach far owing to their size (Bailey, 1966; Heinken, 1999; Büdel & Scheidegger, 2008). Alternatively, they consist of only one symbiont (aposymbiosis), such as hormogonia (cyanobacterial photobionts) or conidia (mycobiont). The haploid lichen‐forming fungus *L. pulmonaria* is a good model of lichen dispersal because it reproduces both clonally and sexually (Scheidegger & Werth, 2009) and is self‐incompatible (Singh *et al*., 2012, 2015). As a sessile, perennial lichen, *L. pulmonaria* lends itself well to dispersal and population studies. Population studies are difficult in most non‐lichenized ascomycetes, where individuals persist in the form of mycelia, but are visible only when they produce (ephemeral) fruiting bodies. As in many fungi, sexual reproduction by (aposymbiotic) ascospores of the mycobionts requires preliminary mating, and both self‐incompatible and self‐mating systems have been reported (Honegger *et al*., 2004; Honegger & Zippler, 2007). Although not all details of the sexual reproduction of lichen‐forming ascomycetes have been described, it has been hypothesized that typically it follows the same lines as frequently reported for non‐lichenized fungi, that is through the fusion between a small gamete (a spermatium) and a receptive hypha (the trichogyne) (Honegger, 1984; Culberson *et al*., 1988; Zoller *et al*., 1999; Sanders, 2014). After fertilization, dikaryotic hyphae induce the development of fruiting bodies, in which the meiotic ascospores are formed. *L. pulmonaria* produces bacteria‐sized conidia (also called microconidia) (Smith *et al*., 2009). As these structures rarely germinate (Vobis, 1977), they are generally considered as male gametes involved in fertilization (Honegger & Scherrer, 2008).

As lichen‐forming ascomycetes are sessile organisms, the male gametes must be transported from a donor to a recipient individual for fertilization to occur. So far, evidence concerning the dispersal distances of lichen (and ascomycete) gametes is limited. Two studies found short‐distance dispersal over centimetres (Culberson *et al*., 1988, 1993), and a third reported distances over a hundred metres (Keller & Scheidegger, 2016). Two studies have reported dispersal distances for male gametes in ascomycetes. In truffles, the dispersal distance of male gametes is extremely short, and matings mainly occur among offspring from the same fruiting body (Taschen *et al*., 2016). In a wood‐decaying ascomycete, male gametes dispersed over distances up to 36 m (Guidot *et al*., 2003). The male gametes in lichens form a slimy mass oozing out from the producing structure (pycnidia) during wet conditions. As this slimy mass does not appear to lend itself to wind dispersal, male gametes have been hypothesized to be dispersed by rain‐splash (Culberson *et al*., 1988; Honegger & Scherrer, 2008), insectivorous birds, squirrels or invertebrate species, such as hymenopteran and dipteran insects or slugs (Keller & Scheidegger, 2016).

**Figure 1:**
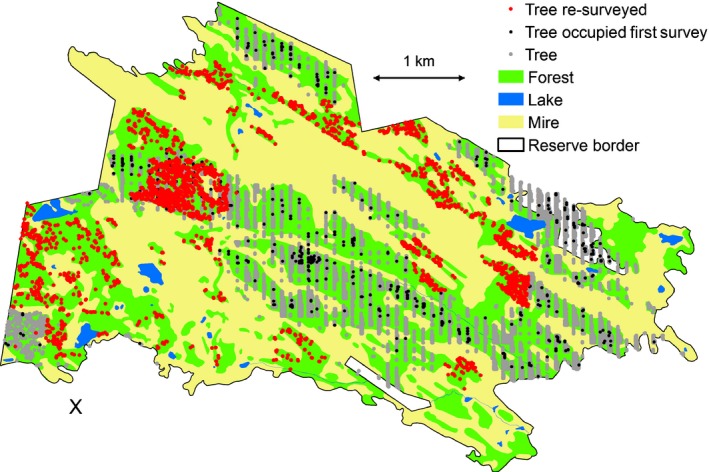
Trees mapped in the first and second surveys in the studied old‐growth landscape in Kuhmo, Finland. Grey dots represent trees non‐occupied and black dots represent trees occupied with *Lobaria pulmonaria* in the first survey. Red dots represent trees re‐surveyed. The coordinate of the reference point ‘X’ is 63.881°N, 29.187°E.

The aim of this study was to fit the mathematical functions describing the dispersal of clonal propagules, gametes and ascospores of the lichen‐forming ascomycete *L. pulmonaria* (Peltigerales). We used the hierarchical Bayesian parentage analyses proposed by Moran & Clark (2011) which integrate genetic and ecological data. This joint estimation of dispersal range for the three dispersal units was based on 37 colonization events detected on trees re‐surveyed 10 yr after an initial complete survey of the species in a 2‐km^2^ landscape (Fig. 1).

## Materials and Methods

### Model species and field sampling

The basis for the study was a complete survey of aspen (*Populus tremula* L.) and goat willow (*Salix caprea* L.) trees (*n *=* *11 038) performed in a landscape consisting of *c*. 20 km^2^ of old‐growth forest in Kuhmo, eastern Finland, during 1997–1999 (Fig. 1). Aspen and goat willow are host trees of *Lobaria pulmonaria* L. (Hoffm.) in the region. The presence/absence of *L*.* pulmonaria* was recorded on these 11 038 host trees (Gu *et al*., 2001). During 2007–2010, two further surveys were conducted. First, we re‐surveyed a subset of 3085 host trees (red dots, Fig. 1) that were unoccupied in the first survey, aiming to find offspring colonization events, that is trees unoccupied in the first survey and occupied in the second survey. During the 10‐yr period, 37 trees had become colonized by a total of 46 offspring thalli. We collected samples from each of these 46 thalli. Second, we re‐surveyed the 594 potential dispersal sources, that is the trees that were found to be occupied in the first survey (black dots, Fig. 1). These were spread out across the entire study landscape (Fig. 1). On each of these 594 trees, we sampled thalli after recording the number of individual thalli and their total cover. Up to three thallus samples were collected from each tree. If there were three or more thalli, we sampled the largest one, one thallus situated directly below it and one on the opposite side of the trunk. By sampling below, we aimed to maximize the chance of capturing reproduction within the tree as diaspores probably often pour down the stem. Samples taken from the opposite side of the tree aimed to maximize the chance of sampling immigration events as soredia/isidia produced by local thalli probably more often establish on the same side than on the opposite side of the stem. The total number of samples was 1131, collected from 631 trees (37 newly colonized plus 594 dispersal sources).

### DNA extraction, microsatellite genotyping and population‐genetic analyses

Pieces of the lobe tips of *L. pulmonaria*, one to two square centimetres in size, were torn to small fragments and lyophilized. DNA was extracted from the fragments using a Qiagen DNeasy Plant Kit (Qiagen, Solna, Sweden) following the manufacturer's instructions. All samples were genotyped at the following 11 fungal microsatellite loci specific to *L*. *pulmonaria*: LPu09, LPu15, LPu23, LPu25, LPu28 (Walser *et al*., 2003), LPu37451, LPu04843, LPu17457, LPu39912, LPu13707 (Werth *et al*., 2013) and MS4 (Dal Grande *et al*., 2012). Two multiplex polymerase chain reactions (PCRs) were performed in a reaction volume of 5 μl each. The first multiplex PCR contained 0.5 μl genomic DNA (*c*. 10–50 ng), 100 nM of the primer pair LPu28, 200 nM of the primer pair LPu37451, 250 nM of the primer pair LPu09, 300 nM of the primer pair LPu25, 500 nM of the primer pair MS4, as well as 1.5 μl nuclease‐free H_2_O, 2.5 μl TypeIT Microsatellite PCR Master Mix and TE buffer (0.73 mM Tris·Cl, 0.073 mM EDTA; Qiagen). The second multiplex included 100 nM of the primer pairs LPu17457, LPu39912 and LPu13707, 200 nM of the primer pairs LPu23 and LPu04843, and 350 nM of the primer pair LPu15. We used the fluorescent labels VIC, NED, PET and FAM to separate the loci within each multiplex. The amplification protocol for both multiplex amplification reactions began with denaturation at 95°C for 5 min for activation of the hot‐start enzyme, followed by 28 cycles of denaturation at 95°C for 30 s, annealing at 57°C for 90 s and extension at 72°C for 30 s. A final extension followed at 60°C for 30 min. All amplification was performed with a thermal cycler (GeneAmp PCR System 2700, Applied Biosystems, Vienna, Austria and Nexus Gradient, Eppendorf). The fragment lengths of the PCR products were determined using a 3730 DNA Analyzer (Applied Biosystems). Genotyping was performed using PeakScanner v.2 (Applied Biosystems, Stockholm, Sweden) and manual editing. One of the 11 loci included in multiplex PCRs, LPu39912, was excluded from further analyses as it was closely linked to LPu04843. Two additional loci were excluded because they did not amplify (LPu37451) or were not polymorphic (LPu23). Therefore, the analyses are based on eight polymorphic microsatellite loci. To ensure correct scoring, 8698 alleles were scored twice. Any alleles that were scored to different lengths were scored again and corrected. The resulting data are stored at ECDS with the metadata standard name ISO 19115:2003 ECDS and file identifier 0c792f24‐6df9‐4a24‐a447‐9e43156b4250.

The number of alleles per locus was calculated with Genepop 4.2 (Rousset, 2008). Nei's unbiased gene diversity (Nei, 1978) was calculated with Arlequin 3.1 (Excoffier *et al*., 2005).

### Bayesian estimation of dispersal functions

#### Datasets

Parentage analyses with Bayesian inference were used to estimate three effective dispersal functions involved in the reproduction and dispersal of *L. pulmonaria*. We estimated the effective dispersal distances of gametes between parents and between maternal individuals and offspring for both sexual ascospores and clonal propagules.

To ensure the robustness of our results, several different approaches were taken to analyse the data. For cases in which several samples were taken from the same tree, we used two different approaches. As a result of clonal dispersal, numerous genetically identical thalli are often found on the same tree. For this reason, in the first approach, we considered samples from the same tree as different individuals if their genotypes were different, and as a single individual if their genotypes were identical. In one analysis, we dropped the clones of the same individual which reduced the number of samples. In this process, 219 clones were dropped. However, there is a small chance that individuals which have identical alleles for the analysed markers are not entirely genetically identical, and could therefore possibly reproduce sexually. Therefore, in our second approach, we considered samples from the same tree as different individuals even if their genotypes were identical – the clone samples were not dropped. These approaches are termed ‘clones dropped’ and ‘clones not dropped’ in the Supporting Information.

Some of the colonization events by offspring may represent potential parent thalli that were not detected in the first survey (Belinchón *et al*., 2017), and ignoring this could bias our dispersal range estimates. We therefore analysed the data in three different ways (see Supporting Information): In analysis I, with 46 offspring and 866 potential parent thalli among a total of 631 trees, we classified all thalli on trees which were unoccupied by *L*.* pulmonaria* in the first survey, but colonized in the second survey, as offspring. However, it is also well known that a proportion of occupied trees are erroneously recorded as unoccupied, 4.4% in this study system (Belinchón *et al*., 2017). In analyses II and III, we accounted for imperfect detection and classified some of these individuals as potential parents instead of offspring. In analysis II, we reclassified some of the 46 offspring as, instead, potential parent thalli; we specifically reclassified colonized trees with more than one thallus and trees with a total thallus cover higher than 50 cm^2^ as potential parents. This dataset consisted of 13 offspring and 899 parent thalli. In analysis III, we ignored whether the trees had been colonized between the two surveys. We only used the total cover and number of *L*. *pulmonaria* thalli on each tree as a basis for the determination of whether an individual was an offspring or a potential parent. Specifically, trees with a maximum of one thallus and cover of < 40 cm^2^ were classified as offspring, whereas all thalli on trees with > 40 cm^2^ of cover or more than one thallus were considered as potential parents. In addition, thalli on trees which were dead or had < 100% bark cover were never considered as offspring as there is a risk of thalli falling off from trees when the bark begins to fall off or decompose. This can result in only a small thallus remaining on a tree. The analysis III data consisted of 774 potential parent thalli and 24 offspring. In addition to the 219 dropped clones, 114 samples were removed from the dataset. The choice of the 40‐ and 50‐cm^2^ thresholds was based on the plotted distributions of thalli cover and thalli numbers per tree (not shown). We also compared the number of colonization events with the colonization rate estimated for a nearby landscape (Belinchón *et al*., 2017); the choice of a thallus cover that was too low resulted in unrealistically few colonization events and of one that was too high resulted in unrealistically many colonization events.

#### Dispersal models fitted

We analysed the data using a modelling approach similar to that of Moran & Clark (2011) but tailored to the current system. The aim was to fit the dispersal function of the species, that is the probability that the dispersal unit deposits at a location at least at distance *x* from its source. This is not the probability of establishment. The dispersal function also allowed us to present the probability density function (PDF), that is the probability that a dispersal unit deposits between any two distance values. This probability is given by the integral of the PDF between these two values.

In this model, we assumed that each offspring resulted from either clonal propagation (with probability *c*) or sexual reproduction (with probability 1 − *c*). The possibility of selfing was excluded, as this does not occur in the self‐incompatible model species. As a heterothallic ascomycete, *L. pulmonaria* is hermaphroditic and bears two mating types, and individuals of one mating type can only mate with those of the opposite type (Singh *et al*., 2012). We assumed that any two individuals from the parent generation were equally likely to represent compatible mating types. For our study area, the spatial distribution of mating types in *L. pulmonaria* was unknown. In fact, the mating types do not influence parameter estimation, as all pairs of parents have the same probability of being of compatible type. This is true even in the case of skewed frequencies of mating types, as long as they are evenly distributed in space in the same skewed ratio. However, local clonal dispersal is likely to lead to spatial aggregation of mating types, and this has indeed been reported for *L. pulmonaria* (Singh *et al*., 2015). With regard to our model, such spatial aggregation would mean that we would underestimate the effective dispersal range of male gametes, because individuals of opposite mating type would be further away from each other, on average, than expected under a random spatial distribution. Thus, our estimate of the dispersal range of male gametes was conservative and the range could thus be longer.

We indexed the offspring using *o*. We denoted by $p_{mf}^{o}$ the probability that the genotype of the individual *o* would result from reproduction between a potential maternal *m* and a potential paternal individual *f*. To calculate *p*_*mf*_ , we denoted the measured genotypes for the offspring, maternal and paternal individuals for locus *i* by *A*_*oi*_, *A*_*mi*_ and *A*_*fi*_ , respectively. We denoted by $q_{mi}^{o}$ and $q_{fi}^{o}$ the probabilities that the maternal individual *m* and the paternal individual *f* had the same microsatellite multilocus genotype in locus *i* as the offspring genotype. To compute $q_{mi}^{o}$, we denoted the genotyping error probability by ɛ, and noted that there are the following four possibilities:

1. The maternal individual and the offspring are genotyped correctly, with probability (1 − ɛ)^2^ = 1 − 2ɛ + ɛ^2^.
2. The maternal genotype is correct, but the offspring genotype is not, with probability ɛ(1 − ɛ) = ɛ − ɛ^2^.
3. The offspring genotype is correct, but the maternal genotype is not, with probability ɛ(1 − ɛ) = ɛ − ɛ^2^.
4. The offspring and maternal genotypes are both erroneous, with probability ɛ^2^.

If an allele was genotyped erroneously, or if it was not genotyped at all, we assumed that the true allele was *A* with probability *f* (*A*), which stands for the frequency of the allele *A* as observed in the entire population. Accordingly, $q_{mi}^{o}$ was computed as follows:

1. If *A*_*mi*_ ≠ *A*_*oi*_, then $q_{mi}^{o}=\left(\varepsilon -\varepsilon^{2}\right)\left[f\left(A_{oi}\right)+f\left(A_{mi}\right)\right]+\varepsilon^{2}z$ where *z* = ∑ _*A*_ *f*(*A*)^2^ is the probability that two alleles sampled randomly from the population are equal.
2. If *A*_*mi*_ = *A*_*oi*_, then $q_{mi}^{o}={\left(1-\varepsilon \right)}^{2}+\varepsilon^{2}z$.
3. If the maternal individual was not genotyped for the gene, we have $q_{mi}^{o}=\left(1-\varepsilon \right)f\left(A_{oi}\right)+\varepsilon z$.
4. If the offspring was not genotyped for the gene, we have $q_{mi}^{o}=\left(1-\varepsilon \right)f\left(A_{mi}\right)+\varepsilon z$.
5. If neither the offspring nor the maternal individual was genotyped for the gene, we have $q_{mi}^{o}=z$.

As the probability of inheriting each locus from either parent is equal, we have $p_{mf}^{o}=\prod_{i}^{n_{g}}\left(q_{mi}^{o}+q_{fi}^{o}\right)/2$, where the probability $q_{fi}^{o}$ can be computed identically as $q_{mi}^{o}$. As selfing was excluded, we set $p_{mf}^{o}=0\;\text{for}\;m=f$.

We denoted the effective ascospore dispersal function by *z* ^*M*^(*d*) and the male gamete dispersal function by *z* ^*S*^(*d*), where *d* is distance. We denoted the location‐based probability by which an offspring arose specifically from maternal *m* and paternal *f* by $r_{mf}^{o}$. As the mating takes place at the maternal individual's host tree, we have

$$r_{mf}^{o}=\frac{z^{M}\left(d_{om}\right)}{\tau_{M}z^{M}\left(0\right)+\sum_{m^{\prime }}z^{M}\left(d_{om^{\prime }}\right)}\times \frac{z^{s}\left(d_{mf}\right)}{\tau_{F}z^{S}\left(0\right)+\sum_{f^{\prime }}z^{S}\left(d_{mf^{\prime }}\right)},$$

for *m* ≠ *f* and otherwise $r_{mf}^{o}=0$. Here, *d*_*om*_ is the distance between the offspring *o* and maternal individual *m*, and *d*_*mf*_ is the distance between the maternal *m* and the maternal individual *f*. The parameters τ_*M*_ and τ_*F*_ measured the effective numbers of maternal and paternal individuals not included in the sampled population. Denoting the cases of missing maternal and paternal individuals by m* and *f*  *, respectively, we have

$$r_{m^{*}f}^{o}=\frac{\tau_{M}z^{M}\left(0\right)}{\tau_{M}z^{M}\left(0\right)+\sum_{m^{\prime }}z^{M}\left(d_{om^{\prime }}\right)}\times \frac{z^{S}\left(d_{mf}\right)}{\tau_{F}z^{S}\left(0\right)+\sum_{f^{\prime }}z^{S}\left(d_{mf^{\prime }}\right)},$$

$$r_{mf^{*}}^{o}=\frac{z^{M}\left(d_{om}\right)}{\tau_{M}z^{M}\left(0\right)+\sum_{m^{\prime }}z^{M}\left(d_{om^{\prime }}\right)}\times \frac{\tau_{F}z^{S}\left(0\right)}{\tau_{F}z^{S}\left(0\right)+\sum_{f^{\prime }}z^{S}\left(d_{mf^{\prime }}\right)},$$

$$r_{m^{*}f^{*}}^{o}=\frac{\tau_{M}z^{M}\left(0\right)}{\tau_{M}z^{M}\left(0\right)+\sum_{m^{\prime }}z^{M}\left(d_{om^{\prime }}\right)}\times \frac{\tau_{F}z^{S}\left(0\right)}{\tau_{F}z^{S}\left(0\right)+\sum_{f^{\prime }}z^{S}\left(d_{mf^{\prime }}\right)}.$$

It should be noted that, as we normalized the male gamete dispersal function, it is assumed that there is no male gamete limitation, in the sense that the probability of sexual reproduction did not depend on the availability of potential paternal individuals.

#### Clonal reproduction

We computed the probability of the offspring *o* arising clonally from parent *p* as $p_{p}^{o}=\prod_{i}^{n_{g}}q_{pi}^{o}$, where $q_{pi}^{o}$ is computed as explained above for the potential maternal and paternal individuals. We denoted the clonal dispersal function by *z* ^*C*^(*d*), and denoted the location‐based probability by which an offspring was reproduced by parent *p* by $r_{p}^{o}$. We assumed that

$$r_{p}^{o}=\frac{z^{C}\left(d_{op}\right)}{\tau_{C}z^{C}\left(0\right)+\sum_{p^{\prime }}z^{C}\left(d_{op^{\prime }}\right)},$$

$$r_{p^{*}}^{o}=\frac{\tau_{C}z^{C}\left(0\right)}{\tau_{C}z^{C}\left(0\right)+\sum_{p^{\prime }}z^{C}\left(d_{op^{\prime }}\right)},$$

where the parameter τ_*C*_ was related to the number of missing clonal parents.

#### Dispersal functions

Based on the above definitions, the effective dispersal functions *z* ^*M*^(*d*), *z* ^*S*^(*d*) and *z* ^*C*^(*d*) model the density of a viable propagule depositing at a specific location at distance *d*. We denoted by *Z* ^*M*^(*d*), *Z* ^*S*^(*d*) and *Z* ^*C*^(*d*) dispersal functions that measure the density of propagules deposited to any location at distance *d*. These two are connected by *Z*(*d*) = *z*(*d*)2*πd*, and they were normalized as $\int_{0}^{\infty }\;Z\left(d\right)\;dd=\int_{0}^{\infty }z\left(d\right)2\pi d\;dd=1$ To allow for the possibility of both short‐ and long‐distance dispersal, we modelled all dispersal functions *z*(*d*) using the bivariate Student *t* distribution with scale matrix *α* ^2^ **I** and shape parameter ν. Thus, we set the dispersal functions *z* ^*M*^(*d*), *z* ^*S*^(*d*) and *z* ^*C*^(*d*) to the probability density of a bivariate Student *t* distribution at location (*x*,* y*), where $d=\sqrt{x^{2}+y^{2}}$. We estimated the scale and shape parameters, denoted by *a*_*M*_, *a*_*S*_ and *a*_*C*_, and ν_*M*_, ν_*S*_ and ν_*C*_, respectively.

#### Likelihood of data

The likelihood of observing the genotypic data for the individual *o* is given by

$$L_{o}=c\sum_{p}r_{p}^{o}p_{p}^{o}+\left(1-c\right)\sum_{m,f}r_{mf}^{o}p_{mf}^{o},$$

and thus the total likelihood of the data by *L* = ∏ _*o*_ *L*_*o*_. Above, the sums also include the cases of missing individuals (p*, m* and *f* *), for which the genotypes were assumed to be unknown.

#### Parameter estimation

The parameter vector to be estimated is θ = (*c*, *a*_*M*_, *a*_*S*_, *a*_*C*_, ν_*M*_, ν_*S*_, ν_*C*_, τ_*M*_, τ_*S*_, τ_*C*_). We logit transformed the parameter *c* and log‐transformed the other parameters, and defined for each transformed parameter the *N*(0,10)^2^ prior distribution. In addition, we truncated the prior (in order to avoid numerical problems in the computation of the likelihood) so that the scale parameters *a*_*M*_, *a*_*F*_ and *a*_*C*_ were restricted to the range from 10 m to 10 km, and the shape parameters ν_*M*_, ν_*F*_ and ν_*C*_ were restricted to the range from 1 to 10. The range for the scale parameter reflects the scale of dispersal distances that can be estimated given the study design, whereas the range for the shape parameter allowed for both thin‐ and fat‐tailed dispersal functions. We sampled the posterior distribution with the single‐component Accept algorithm (Ovaskainen *et al*., 2008).

## Results

### Dispersal range, proportion of clonal reproduction and genetic diversity

**Figure 2:**
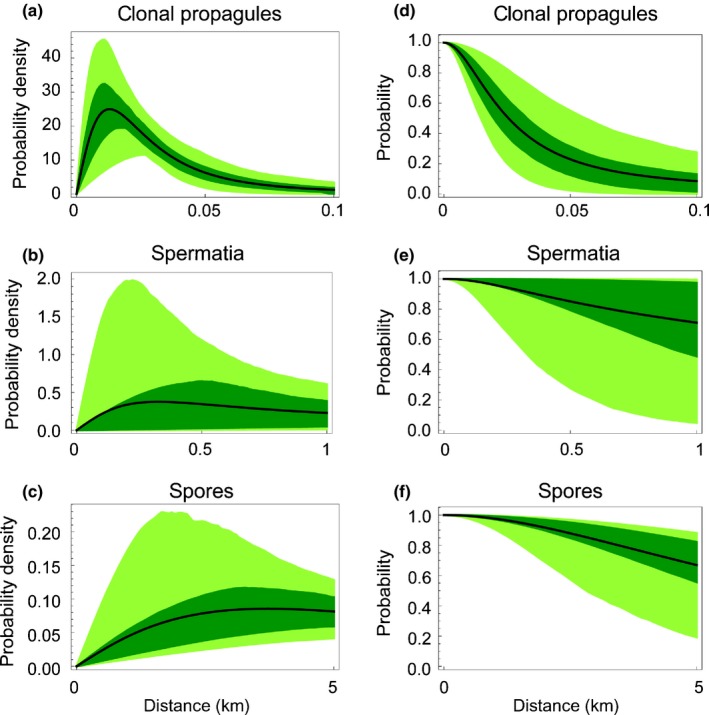
Effective dispersal distance and probability of deposition to at least distance *x* for clonal propagules, spermatia and spores in the epiphytic lichen‐forming ascomycete *Lobaria pulmonaria*. (a–c) The probability density function (PDF; × 10^3^) for deposition, that is the probability that a dispersal unit deposits between any two distance values. This probability is given by the integral of the PDF between these two values. (d–f) The dispersal functions, that is the probability that the dispersal unit deposits at a location at least at distance *x* from its source. Black lines show the posterior medians, and the shading shows the upper and lower 25% (dark green) and 2.5% (light green) posterior quantiles. Note the different scaling of the *x*‐axes. The figure represents analysis I, clones not dropped (see the Materials and Methods section).

We observed 37 new *L. pulmonaria* colonization events that had taken place during the 10‐yr period, involving 46 thalli. These resulted from distinctly different effective dispersal ranges of the three types of dispersal units, as revealed by the statistical modelling of the genetic data. The effective median dispersal range of clonal symbiotic propagules was 26 m, but the most common distance (the mode) was 10 m and the probability of clonal dispersal further than 100 m was only 8% (Fig. 2; Table 1). By contrast, the effective dispersal range of the two aposymbiotic diaspore types was considerably longer (Fig. 2). For male gametes, the most common dispersal range was 100 m (the mode, Fig. 2), the median was 3 km and the probability of dispersal further than 5 km was 23% (Table 1). The ascospore dispersal range was even further and we could essentially not establish any restricted dispersal within the study landscape. The median ascospore dispersal range was 8.1 km and the probability of dispersal beyond 5 km (the width of the study landscape; Fig. 1) was 67%. We also show uncertainties in these summary estimators (Fig. 2, Table 1), resulting from the uncertainties in the estimates of the parameters of the dispersal models for the different dispersal units (Table 2). These results were robust and did not depend on the modelling criteria used; with all methods, we found long‐distance dispersal of male gametes and ascospores and short‐distance dispersal of clonal propagules (Supporting Information Figs S1–S5).

**Table 1:** Summary statistics (mean and Bayesian 95% credible intervals) on dispersal ranges (in metres) of clonal propagules, gametes and ascospores of *Lobaria pulmonaria* over a 10‐yr period

	Clonal propagules	Gametes	Ascospores
Median distance	26 (14, 53)	2950 (200, 12 019)	2950 (2704, 14 605)
Mean distance	48 (17, 121)	919 (266, 2037)	919 (378, 2029)
*P*(10 m)^a^	0.84 (0.68, 0.96)	1 (1, 1)	1 (1, 1)
*P*(100 m)^a^	0.08 (0, 0.26)	0.96 (0.81, 1)	0.96 (1, 1)
*P*(1000 m)^a^	3.9 × 10^−3^ (1.6 × 10^−11^, 2.1 × 10^−2^)	0.52 (0.0059, 0.99)	0.52 (0.89, 0.99)
*P*(5000 m)^a^	6.0 × 10^−4^ (2.2 × 10^−16^, 3.9 × 10^−3^)	0.23 (8.5 × 10^−8^, 0.86)	0.23 (0.19, 0.88)

a *P*(*x*) refers to the probability that the dispersal unit deposits at least *x* m away from a dispersal source.

**Table 2:** Estimates of the parameters (median and Bayesian 95% credible intervals) of the dispersal functions for clonal propagules (*C*), gametes (*S*) and ascospores (*M*) of *Lobaria pulmonaria*

Parameter	Estimate
*a*_*C*_ a	17.8 (10.5, 38.0)
*a*_*S*_ a	1868 (242, 9146)
*a*_*M*_ a	5800 (2159, 9745)
ν_*C*_ a	1.95 (1.03, 9.02)
ν_*S*_ a	3.03 (1.06, 9.43)
ν_*M*_ a	3.73 (1.08, 9.58)
τ_*C*_ b	2.7 × 10^−5^ (6.5 × 10^−11^, 3.3 × 10^−2^)
τ_*S*_ b	6.0 × 10^−3^ (2.1 × 10^−9^, 10.3)
τ_*M*_ b	5.7 × 10^−3^ (2.2 × 10^−10^, 16.4)

a Scale (*a*) and shape (ν) parameters of the dispersal function.

b τ measures the effective numbers of clonal parents and paternal and maternal individuals not included in the sampled population.

The posterior median estimate (95% credible interval) for the proportion of clonal reproduction was *c *=* *0.38 (0.25–0.53). Thus, sexual reproduction took place at a frequency of 1 – 0.38 = 0.62. Nei's unbiased gene diversity was 0.55 (standard error 0.30).

### Polymorphism and genotyping error rate

The eight loci selected for further analyses were highly polymorphic with, on average, 26 alleles per locus, and a range of seven to 78 alleles (Table 3).

**Table 3:** Information from eight microsatellite loci for the lichen‐forming ascomycete *Lobaria pulmonaria* in the study area (Fig. 1)

Locus	Total sample size	Number of alleles	Gene diversity
LPu04843	1044	78	0.83
LPu09	1024	51	0.93
LPu13707	1148	9	0.58
LPu15	1145	14	0.43
LPu17457	1148	11	0.26
LPu25	1138	31	0.83
LPu28	1135	7	0.61
MS4	1134	7	0.40
Average	1114.5	26	0.61

Nineteen of the 8698 alleles genotyped twice showed an inconsistent result. The error rates, calculated as the number of alleles with an inconsistent result divided by the total number of alleles for each locus, were as follows 1/1109 (LPu25), 2/1106 (LPu28), 1/1106 (MS4), 9/1000 (LPu09), 1/1118 (LPu15), 3/1017 (LPu04843), 1/1121 (LPu17457) and 1/1121 (LPu13707). Excluding locus LPu09 (with the highest error rate), the genotyping error rate was ɛ = 10/7698 ≈ 0.0013; this was utilized for all loci except locus LPu09. The observed numbers of errors for loci other than LPu09 could have been produced with this error rate, as the PDFs for the binomial distribution with *n* = 1140 are 0.24, 0.34, 0.25 and 0.12 for zero, one, two and four errors, respectively. For locus LPu09, there is evidence for a higher error rate, so we use the locus‐specific estimate ɛ = 9/1000 ≈ 0.009. The error rate is used in the parentage analysis, which takes into account the risk that a sample is genotyped incorrectly.

## Discussion

Here, as far as we are aware, we have formulated the first model to jointly estimate the effective dispersal range for clonal propagules, male gametes and ascospores of a haploid fungus. The effective dispersal range of the relatively large symbiotic propagules of *L. pulmonaria* was typically 10 m and rarely reached further than 100 m. Male gametes dispersed 100–1000 m, and ascospores dispersed several kilometres. Based on size, we would have expected clonal propagules (120 × 100 μm^2^, Werth *et al*., 2014) to disperse the shortest distance, ascospores (18–30 × 5–9 μm^2^, Smith *et al*., 2009) to disperse over an intermediate distance and male gametes (5 × 1 μm^2^, Smith *et al*., 2009) to disperse the longest distance, but, instead, the male gametes dispersed over a shorter distance than the ascospores. The model builds on Moran & Clark (2011) and provides a solid foundation for future studies of effective dispersal in self‐incompatible haploid species. It can also be extended to other haploid organisms.

The robustness of our estimates for the effective dispersal range of male gametes is supported by the agreement between our joint estimates for the dispersal of clonal propagules and ascospores with preceding studies of lichen‐forming fungi. Our estimated clonal dispersal of 10–100 m is confirmed by several studies of lichen‐forming fungi (Tapper, 1976; Werth *et al*., 2006a; Scheidegger & Werth, 2009), including one combining data of 62 populations across Europe, North America, Asia and Africa (Dal Grande *et al*., 2012). Long‐distance dispersal of ascospores at various scales has been hypothesized in various studies of lichenized ascomycetes (Printzen *et al*., 2003; Muñoz *et al*., 2004; Geml *et al*., 2012; de Paz *et al*., 2012; Leavitt *et al*., 2013; Bendiksby *et al*., 2014), and the importance of ascospores for the colonization of new substrates has also been emphasized in non‐lichenized ascomycetes (Johannesson *et al*., 2001; Fraaije *et al*., 2005) and ectomycorrhizal fungi (reviewed in Douhan *et al*., 2011). Ascospores disperse over long distances by wind (Gjerde *et al*., 2015; Alors *et al*., 2017) as they are of microscopic size and actively discharged. Rieux *et al*. (2014) inferred the effective dispersal distances of ascospores (size 11.5–16.5 × 2.5–5.0 μm^2^; www.cabi.org/isc/datasheet/35278) and conidia (size 30–132 × 2.5–5 μm^2^) of a plant pathogen using spore trap plants. Similar to our results, they found long‐distance ascospore dispersal (up to 1000 m), as well as local dispersal of the large conidia (up to 12.5 m). In the plant pathogen *Zymoseptoria tritici* (syn. *Mycosphaerella graminicola*), rain‐splash‐dispersed large multicellular conidia (size 40–100 × 1.5–3.5 μm^2^, Steinberg, 2015) ranged over only a few metres, whereas ascospores (size 10–15 × 2–3 μm, Steinberg, 2015) dispersed up to 85 m (Fraaije *et al*., 2005). It can be assumed that short‐distance dispersal of locally selected clones facilitates a high rate of local population establishment, whereas recombinant genotypes that are dispersed over long distances are better adapted to increasingly changing habitat conditions. A combined reproduction strategy that includes both clonal and sexual reproduction underlies the success of many, but not all, clonal plant species (Vallejo‐Marìn *et al*., 2010) and fungi.

Few studies have reported dispersal distances for male gametes in lichen‐forming fungi. Two studies based on the chemotyping of single ascospore progeny found effective short‐distance dispersal of gametes (Culberson *et al*., 1988, 1993). Keller & Scheidegger (2016) assigned paternities to single ascospores of *L. pulmonaria* and found dispersal distances of male gametes over 100 m. Here, we show that male gametes may frequently disperse over several hundred metres, up to kilometres. Effective dispersal of male gametes over a comparatively large distance (36 m) has been reported previously for a non‐lichenized saprotrophic ascomycete based on parentage assignments (Guidot *et al*., 2003).

Although it has been speculated that the male gametes of lichenized fungi may be dispersed either by rain splash or via insects (Honegger & Scherrer, 2008), the vectors mediating gamete dispersal remain unknown. That the male gametes dispersed over a shorter distance than ascospores implies that they are not wind dispersed. Our estimate of effective gamete dispersal for lichen‐forming ascomycetes is of the same order of magnitude as, or even higher than, that which has been reported for insect‐ or wind‐pollinated plants (Austerlitz *et al*., 2004). Thus, the male gametes of lichenized fungi are transmitted by vectors that are as effective as pollen dispersers in plants. There is at least one example of insect‐mediated dispersal of male gametes in non‐lichenized ascomycetes: the grass endophyte *Epichloë elymi*. The anthomyiid fly *Phorbia phrenione* lays eggs on the fungal fruiting body, which larvae partly eat, and mediates male gametes between fungal individuals to ensure fruiting body formation (Bultman & White, 1988). It is unlikely that slugs feeding on lichens, such as *L. pulmonaria* (Coker, 1967; Boch *et al*., 2011), act as important dispersal vectors of male gametes over several hundreds of metres. Instead, the vectors of male gametes could include hymenopteran and dipteran insects or insectivorous birds that are visiting *L. pulmonaria* thalli in search of prey (Keller & Scheidegger, 2016). Although our analysis does not identify a specific type of male gamete, soredia, the sole clonal propagules in *L. pulmonaria*, are not likely to act as male gametes because they disperse over much shorter distances (within 100 m) than the distances we observed for gametes. In the lichen *Cladonia furcata*, microconidia have been found to adhere to and fuse with lichen trichogynes, which points towards their role as male gametes (Honegger, 1984). Although the transfer of a nucleus from a microconidium to a trichogyne has not yet been proven experimentally in lichenized fungi, microconidia are nevertheless the most likely fertilizing agent in these fungi. However, our data do not allow us to exclude the long‐ranging ascospores as fertilizing agents. For truffles, ascospores are suspected as male gametes (Taschen *et al*., 2016), but they do not disperse as far as the ascospores of *L. pulmonaria*.

Our results of long‐distance effective dispersal of male gametes imply that mating is possible among distantly located individuals in old‐growth forests (Fig. 1), which should lead to high genetic variability (Williams, 1994). High variability and a high proportion of colonizations originating from (recombined) ascospores were indeed found in the old‐growth forest, similar to reports in other studies (Werth *et al*., 2006b; Hilmo *et al*., 2012; Nadyeina *et al*., 2014). On the contrary, in heavily managed forests, local population densities are often low, distances amongst host trees are large (Gu *et al*., 2001) and short‐distance dispersal dominates (Belinchón *et al*., 2017). Under such conditions, individuals might suffer from male gamete limitation, potentially impeding sexual reproduction, analogous to pollen limitation reported in plants (Ashman *et al*., 2004). The hypothesis of male gamete limitation could be tested in the future by transplanting individuals possessing the structures producing male gametes (pycnidia) in the vicinity of potential partners of compatible mating type. Indeed, genetic diversity has also been found to be lower in heavily managed than in old‐growth forests (Jüriado *et al*., 2011; Scheidegger *et al*., 2012), but is dependent on the type of disturbance (Wagner *et al*., 2006; Werth *et al*., 2006b).

Our results show that symbiotic clonal dispersal operates locally, allowing dispersal mainly amongst neighbouring trees. Clonal dispersal is an important process leading to local population growth and, by the spread to neighbouring trees, the chances of the survival and dispersal of a genotype are increased (Scheidegger & Werth, 2009). This process also leads to local patches of clones, a pattern which has been observed for *L. pulmonaria* (Werth *et al*., 2006b; Singh *et al*., 2015). If the lichen mainly reproduces clonally, genetic patterns of symbionts match, reflecting the predominant co‐dispersal of the lichen's symbionts in symbiotic clonal propagules (Dal Grande *et al*., 2012). Such matching patterns have been reported for the Swiss Jura (Werth & Scheidegger, 2012).

We found that ascospores disperse over long distances, and a high proportion of the juveniles we investigated resulted from sexual processes. A high proportion of sexual reproduction generating far‐dispersing ascospores implies that this lichen‐forming ascomycete is able to disperse over long distances in old‐growth landscapes, supporting earlier findings (Werth *et al*., 2006a; Gjerde *et al*., 2015). It also indicates the potential for the genetic patterns among symbionts to become untangled, because sexual reproduction is associated with horizontal transmission of the green algal photobiont, which so far has not been found in free‐living populations (Dal Grande *et al*., 2014; Werth & Scheidegger, 2012; C. Scheidegger *et al.,* unpublished).

Although the lichen can potentially spread over long distances with long‐ranging ascospores, its spread across the landscape is nevertheless limited by various factors. First, the absolute number of offspring recruited onto thousands of trees within the observed decade was low, implying that establishment limitation may prevent the successful colonization of new trees. This could be because germinating spores may have difficulties in finding compatible photobionts (Werth *et al*., 2006a). Moreover, low effective dispersal in *L. pulmonaria* has been associated previously with establishment limitation (Werth *et al*., 2006a). In managed landscapes with long distances between the trees, both establishment limitation and restricted dispersal range have been found to limit future metapopulation development (Belinchón *et al*., 2017).

Our findings contribute substantially to the understanding of dispersal and reproduction in lichenized ascomycetes at large spatial scales, but also raise the question of how other components of the lichen symbiosis are dispersed, such as the photobiont. The lichen‐forming ascomycete *L. pulmonaria* is exclusively associated with the green algal photobiont *Symbiochloris reticulata* (Dal Grande *et al*., 2014; Škaloud *et al*., 2016). In our study area, the photobiont cannot be taken from other coexisting lichen species. The only method of dispersal of this photobiont is hypothesized to be through symbiotic lichen propagules, stressing the importance of complex photobiont‐mediated lichen guilds (Rikkinen *et al*., 2002; O'Brien *et al*., 2013; Dal Grande *et al*., 2014). Hence, the lichen spread should be limited by the predominant short‐distance dispersal of the photobiont in clonal propagules, despite the potential for long‐distance dispersal of the fungal symbiont through ascospores and male gamete‐mediated gene flow. Here, however, we present evidence that this lichenized fungus established from ascospores dispersed over long distances. As the ascospores only contain the fungal symbiont, the symbiosis was re‐established either by an association with free‐living algal individuals or by obtaining photobionts from soredia dispersed over short distances. The photobiont and mycobiont in *L. pulmonaria* thus reveal contrasting dispersal distances, which can lead to distinct (genetic) pattern formation for the two symbionts (Marco *et al*., 2011), although in a large, continuous population in which the species predominantly disperses vegetatively, congruent genetic patterns have been found amongst the symbionts (Werth & Scheidegger, 2012). The data and model presented here substantially increase our understanding of reproductive biology in the lichen symbiosis, whose biology remains poorly understood, even though it serves as one of the textbook examples for mutualistic symbioses. The model further opens up new ground for the study of the dispersal of haploid organisms, a key process for the dynamics and evolution of populations. Work on dispersal is even more important in the anthropogenically fragmented landscapes of our time and for the assessment of the ability of species to expand into new regions with favourable climatic conditions in the future.

## Author contributions

C.R., S.W., O.O., G.V., C.S. and T.S. wrote the paper, and, in addition, C.R. conducted the genetic work, O.O. formulated the model and fitted it to the data, S.W. supervised the genetic work, G.V. organized the field work, and T.S. initiated and led the study.

## Supporting information

Please note: Wiley Blackwell are not responsible for the content or functionality of any Supporting Information supplied by the authors. Any queries (other than missing material) should be directed to the *New Phytologist* Central Office.

**Fig. S1** Effective dispersal distance and probability of deposition to at least distance *x* for clonal propagules, gametes and ascospores in the epiphytic lichen‐forming ascomycete *Lobaria pulmonaria*. The figure represents analysis I, clones dropped.
**Fig. S2** Effective dispersal distance and probability of deposition to at least distance *x* for clonal propagules, gametes and ascospores in the epiphytic lichen‐forming ascomycete *Lobaria pulmonaria* based on analysis II, clones dropped.
**Fig. S3** Effective dispersal distance and probability of deposition to at least distance *x* for clonal propagules, gametes and ascospores in the epiphytic lichen‐forming ascomycete *Lobaria pulmonaria* based on analysis II, clones not dropped.
**Fig. S4** Effective dispersal distance and probability of deposition to at least distance *x* for clonal propagules, gametes and ascospores in the epiphytic lichen‐forming ascomycete *Lobaria pulmonaria* based on analysis III, clones dropped.
**Fig. S5** Effective dispersal distance and probability of deposition to at least distance *x* for clonal propagules, gametes and ascospores in the epiphytic lichen‐forming ascomycete *Lobaria pulmonaria* based on analysis III, clones not dropped.

## References

[nph14714-bib-0001] Alors D , Dal Grande F , Cubas P , Crespo A , Schmitt I , Molina MC , Divakar PK . 2017 Panmixia and dispersal from the Mediterranean Basin to Macaronesian Islands of a macrolichen species. Scientific Reports 7: 40879. 28102303 10.1038/srep40879 PMC5244402

[nph14714-bib-0002] Ashman TL , Knight TM , Steets JA , Amarasekare P , Burd M , Campbell DR , Dudash MR , Johnston MO , Mazer SJ , Mitchell RJ *et al* 2004 Pollen limitation of plant reproduction: ecological and evolutionary causes and consequences. Ecology 85: 2408–2421.

[nph14714-bib-0003] Austerlitz F , Dick CW , Dutech C , Klein EK , Oddou‐Muratorio S , Smouse PE , Sork VL . 2004 Using genetic markers to estimate the pollen dispersal curve. Molecular Ecology 13: 937–954. 15012767 10.1111/j.1365-294x.2004.02100.x

[nph14714-bib-0004] Bailey R . 1966 Studies on the dispersal of lichen soredia. Journal of the Linnean Society (Botany) 59: 479–490.

[nph14714-bib-0005] Belinchón R , Harrison PJ , Mair L , Várkonyi G , Snäll T . 2017 Local epiphyte establishment and future metapopulation dynamics in landscapes with different spatiotemporal properties. Ecology 98: 741–750. 27984632 10.1002/ecy.1686

[nph14714-bib-0007] Bendiksby M , Mazzoni S , Jorgensen MH , Halvorsen R , Holien H . 2014 Combining genetic analyses of archived specimens with distribution modelling to explain the anomalous distribution of the rare lichen *Staurolemma omphalarioides*: long‐distance dispersal or vicariance? Journal of Biogeography 41: 2020–2031.

[nph14714-bib-0008] Bistis GN . 1981 Chemotrophic interactions between trichogynes and conidia of opposite mating‐type in *Neurospora crassa* . Mycologia 73: 959–975.

[nph14714-bib-0009] Boch S , Prati D , Werth S , Rüetschi J , Fischer M . 2011 Lichen endozoochory by snails. PLoS ONE 6: e18770. 21533256 10.1371/journal.pone.0018770 PMC3076439

[nph14714-bib-0010] Büdel B , Scheidegger C . 2008 Thallus morphology and anatomy In: Nash TH, ed. Lichen biology. Cambridge, UK: Cambridge University Press, 40–68.

[nph14714-bib-0011] Bullock JM , Kenward RE , Hails RS . 2002 Dispersal ecology. Cambridge, UK: Cambridge University Press.

[nph14714-bib-0012] Bultman TL , White JF . 1988 “Pollination” of a fungus by a fly. Oecologia 75: 317–319. 28310853 10.1007/BF00378616

[nph14714-bib-0013] Coker PD . 1967 Damage to lichens by gastropods. The Lichenologist 3: 428.

[nph14714-bib-0014] Coppin E , de Renty C , Debuchy R . 2005 The function of the coding sequences for the putative pheromone precursors in *Podospora anserina* is restricted to fertilization. Eukaryotic Cell 4: 407–420. 15701803 10.1128/EC.4.2.407-420.2005 PMC549327

[nph14714-bib-0015] Culberson CF , Culberson WL , Johnson A . 1988 Gene flow in lichens. American Journal of Botany 75: 1135–1139.

[nph14714-bib-0016] Culberson WL , Culberson CF , Johnson A . 1993 Speciation in lichens of the *Ramalina siliquosa* complex (Ascomycotina, Ramalinaceae): gene flow and reproductive isolation. American Journal of Botany 80: 1472–1481.

[nph14714-bib-0017] Dal Grande F , Beck A , Cornejo C , Singh G , Cheenacharoen S , Nelsen MP , Scheidegger C . 2014 Molecular phylogeny and symbiotic selectivity of the green algal genus *Dictyochloropsis* s. l. (Trebouxiophyceae): a polyphyletic and widespread group forming photobiont‐mediated guilds in the lichen family Lobariaceae. New Phytologist 202: 455–470. 24443895 10.1111/nph.12678

[nph14714-bib-0018] Dal Grande F , Widmer I , Wagner HH , Scheidegger C . 2012 Vertical and horizontal photobiont transmission within populations of a lichen symbiosis. Molecular Ecology 21: 3159–3172. 22384938 10.1111/j.1365-294X.2012.05482.x

[nph14714-bib-0019] Debuchy R , Berteaux‐Lecellier V , Silar P . 2010 Mating systems and sexual morphogenesis in ascomycetes In: Borkovitch K, Ebbole D, eds. Cellular and molecular biology of filamentous fungi. Washington, DC, USA: ASM Press, 501–535.

[nph14714-bib-0020] Douhan GW , Vincenot L , Gryta H , Selosse MA . 2011 Population genetics of ectomycorrhizal fungi: from current knowledge to emerging directions. Fungal Biology 115: 569–597. 21724164 10.1016/j.funbio.2011.03.005

[nph14714-bib-0021] Excoffier L , Laval G , Schneider S . 2005 Arlequin (version 3.0): an integrated software package for population genetics data analysis. Evolutionary Bioinformatics Online 1: 47–50. PMC2658868 19325852

[nph14714-bib-0022] Fleissner A , Diamond S , Glass NL . 2009 The *Saccharomyces cerevisiae* PRM1 homolog in *Neurospora crassa* is involved in vegetative and sexual cell fusion events but also has postfertilization functions. Genetics 181: 497–510. 19064710 10.1534/genetics.108.096149 PMC2644943

[nph14714-bib-0023] Fleissner A , Sarkar S , Jacobson DJ , Roca MG , Read ND , Glass NL . 2005 The so locus is required for vegetative cell fusion and postfertilization events in *Neurospora crassa* . Eukaryotic Cell 4: 920–930. 15879526 10.1128/EC.4.5.920-930.2005 PMC1140088

[nph14714-bib-0024] Fraaije BA , Cools HJ , Fountaine J , Lovell DJ , Motteram J , West JS , Lucas JA . 2005 Role of ascospores in further spread of QoI‐resistant cytochrome b alleles (G143A) in field populations of *Mycosphaerella graminicola* . Phytopathology 95: 933–941. 18944416 10.1094/PHYTO-95-0933

[nph14714-bib-0025] Geml J , Kauff F , Brochmann C , Lutzoni F , Laursen GA , Redhead SA , Taylor DL . 2012 Frequent circumarctic and rare transequatorial dispersals in the lichenised agaric genus *Lichenomphalia* (Hygrophoraceae, Basidiomycota). Fungal Biology 116: 388–400. 22385621 10.1016/j.funbio.2011.12.009

[nph14714-bib-0027] Gjerde I , Blom HH , Heegaard E , Sætersdal M . 2015 Lichen colonization patterns show minor effects of dispersal distance at landscape scale. Ecography 38: 939–948.

[nph14714-bib-0029] Gu WD , Kuusinen M , Konttinen T , Hanski I . 2001 Spatial pattern in the occurrence of the lichen *Lobaria pulmonaria* in managed and virgin boreal forests. Ecography 24: 139–150.

[nph14714-bib-0030] Guidot A , Johannesson H , Dahlberg A , Stenlid J . 2003 Parental tracking in the postfire wood decay ascomycete *Daldinia loculata* using highly variable nuclear gene loci. Molecular Ecology 12: 1717–1730. 12803626 10.1046/j.1365-294x.2003.01858.x

[nph14714-bib-0031] Hanski I . 1999 Metapopulation ecology. Oxford, UK: Oxford University Press.

[nph14714-bib-0032] Healy RA , Smith ME , Bonito GM , Pfister DH , Ge ZW , Guevara GG , Williams G , Stafford K , Kumar L , Lee T *et al* 2013 High diversity and widespread occurrence of mitotic spore mats in ectomycorrhizal Pezizales. Molecular Ecology 22: 1717–1732. 23205556 10.1111/mec.12135

[nph14714-bib-0033] Heinken T . 1999 Dispersal patterns of terricolous lichens by thallus fragments. The Lichenologist 31: 603–612.

[nph14714-bib-0034] Hilmo O , Lundemo S , Holien H , Stengrundet K , Stenøien HK . 2012 Genetic structure in a fragmented Northern Hemisphere rainforest: large effective sizes and high connectivity among populations of the epiphytic lichen *Lobaria pulmonaria* . Molecular Ecology 21: 3250–3265. 22571538 10.1111/j.1365-294X.2012.05605.x

[nph14714-bib-0035] Honegger R . 1984 Scanning electron microscopy of the contact site of conidia and trichogynes in *Cladonia furcata* . The Lichenologist 16: 11–19.

[nph14714-bib-0036] Honegger R , Scherrer S . 2008 Sexual reproduction in lichen‐forming ascomycetes In: Nash TH III, ed. Lichen biology. Cambridge, UK: Cambridge University Press, 94–103.

[nph14714-bib-0037] Honegger R , Zippler U . 2007 Mating systems in representatives of Parmeliaceae, Ramalinaceae and Physciaceae (Lecanoromycetes, lichen‐forming ascomycetes). Mycological Research 111: 424–432. 17512182 10.1016/j.mycres.2007.02.005

[nph14714-bib-0038] Honegger R , Zippler U , Gansner H , Scherrer S . 2004 Mating systems in the genus *Xanthoria* (lichen‐forming ascomycetes). Mycological Research 108: 480–488. 15230000 10.1017/s0953756204009682

[nph14714-bib-0039] Johannesson H , Gustafsson M , Stenlid J . 2001 Local population structure of the wood decay ascomycete *Daldinia loculata* . Mycologia 93: 440–446.

[nph14714-bib-0040] Johansson V , Ranius T , Snäll T . 2012 Epiphyte metapopulation dynamics are explained by species traits, connectivity, and patch dynamics. Ecology 93: 235–241. 22624304 10.1890/11-0760.1

[nph14714-bib-0041] Jüriado I , Liira J , Csencsics D , Widmer I , Adolf C , Kohv K , Scheidegger C . 2011 Dispersal ecology of the endangered woodland lichen *Lobaria pulmonaria* in managed hemiboreal forest landscape. Biodiversity and Conservation 20: 1803–1819.

[nph14714-bib-0042] Keller C , Scheidegger C . 2016 Multiple mating events and spermatia‐mediated gene flow in the lichen‐forming fungus *Lobaria pulmonaria* . Herzogia 29: 435–450.

[nph14714-bib-0044] Kohn LM . 1993 What do we need to know about discomycetous anamorphs? In: Reynolds DR, Taylor JW, eds. The fungal holomorph: mitotic, meiotic and pleomorphic speciation in fungal systematics. Wallingford, UK: CAB International, 129–139.

[nph14714-bib-0045] Kokko H , López‐Sepulcre A . 2006 From individual dispersal to species ranges: perspectives for a changing world. Science 313: 789–791. 16902127 10.1126/science.1128566

[nph14714-bib-0046] Lacey J . 1996 Spore dispersal — its role in ecology and disease: the British contribution to fungal aerobiology. Mycological Research 100: 641–660.

[nph14714-bib-0047] Leavitt SD , Fernandez‐Mendoza F , Perez‐Ortega S , Sohrabi M , Divakar PK , Vondrak J , Lumbsch HT , St Clair LL . 2013 Local representation of global diversity in a cosmopolitan lichen‐forming fungal species complex (Rhizoplaca, Ascomycota). Journal of Biogeography 40: 1792–1806.

[nph14714-bib-0048] Marco DE , Montemurro MA , Cannas SA . 2011 Comparing short and long‐distance dispersal: modelling and field case studies. Ecography 34: 671–682.

[nph14714-bib-0049] Moran EV , Clark JS . 2011 Estimating seed and pollen movement in a monoecious plant: a hierarchical Bayesian approach integrating genetic and ecological data. Molecular Ecology 20: 1248–1262. 21332584 10.1111/j.1365-294X.2011.05019.x

[nph14714-bib-0050] Muñoz J , Felicisimo AM , Cabezas F , Burgaz AR , Martinez I . 2004 Wind as a long‐distance dispersal vehicle in the Southern Hemisphere. Science 304: 1144–1147. 15155945 10.1126/science.1095210

[nph14714-bib-0051] Nadyeina O , Dymytrova L , Naumovych A , Postoyalkin S , Werth S , Cheenacharoen S , Scheidegger C . 2014 Microclimatic differentiation of gene pools in the *Lobaria pulmonaria* symbiosis in a primeval forest landscape. Molecular Ecology 23: 5164–5178. 25244617 10.1111/mec.12928

[nph14714-bib-0052] Nei M . 1978 Estimation of average heterozygosity and genetic distance from a small number of individuals. Genetics 89: 583–590. 17248844 10.1093/genetics/89.3.583 PMC1213855

[nph14714-bib-0053] O'Brien HE , Miadlikowska J , Lutzoni F . 2013 Assessing population structure and host specialization in lichenized cyanobacteria. New Phytologist 198: 557–566. 23406441 10.1111/nph.12165

[nph14714-bib-0054] Ovaskainen O , Rekola H , Meyke E , Arjas E . 2008 Bayesian methods for analyzing movements in heterogeneous landscapes from mark‐recapture data. Ecology 89: 542–554. 18409443 10.1890/07-0443.1

[nph14714-bib-0055] de Paz GA , Cubas P , Crespo A , Elix JA , Lumbsch HT . 2012 Transoceanic dispersal and subsequent diversification on separate continents shaped diversity of the *Xanthoparmelia pulla* group (Ascomycota). PLoS ONE 7: e39683. 22745810 10.1371/journal.pone.0039683 PMC3379998

[nph14714-bib-0056] Printzen C , Ekman S , Tonsberg T . 2003 Phylogeography of *Cavernularia hultenii*: evidence of slow genetic drift in a widely disjunct lichen. Molecular Ecology 12: 1473–1486. 12755876 10.1046/j.1365-294x.2003.01812.x

[nph14714-bib-0057] Rieux A , Soubeyrand S , Bonnot F , Klein EK , Ngando JE , Mehl A , Ravigne V , Carlier J , de Lapeyre de Bellaire L . 2014 Long‐distance wind‐dispersal of spores in a fungal plant pathogen: estimation of anisotropic dispersal kernels from an extensive field experiment. PLoS ONE 9: e103225. 25116080 10.1371/journal.pone.0103225 PMC4130500

[nph14714-bib-0058] Rikkinen J , Oksanen I , Lohtander K . 2002 Lichen guilds share related cyanobacterial symbionts. Science 297: 357. 12130774 10.1126/science.1072961

[nph14714-bib-0059] Rousset F . 2008 GENEPOP'007: a complete re‐implementation of the genepop software for Windows and Linux. Molecular Ecology Resources 8: 103–106. 21585727 10.1111/j.1471-8286.2007.01931.x

[nph14714-bib-0060] Sanders WB . 2014 Complete life cycle of the lichen fungus *Calopadia puiggarii* (Pilocarpaceae, Ascomycetes) documented *in situ*: propagule dispersal, establishment of symbiosis, thallus development, and formation of sexual and asexual reproductive structures. American Journal of Botany 101: 1836–1848. 25366850 10.3732/ajb.1400272

[nph14714-bib-0061] Scheidegger C , Bilovitz PO , Werth S , Widmer I , Mayrhofer H . 2012 Hitchhiking with forests: population genetics of the epiphytic lichen *Lobaria pulmonaria* in primeval and managed forests in Southeastern Europe. Ecology and Evolution 2: 2223–2240. 23139881 10.1002/ece3.341 PMC3488673

[nph14714-bib-0062] Scheidegger C , Werth S . 2009 Conservation strategies for lichens: insights from population biology. Fungal Biology Reviews 23: 55–66.

[nph14714-bib-0063] Singh G , Dal Grande F , Cornejo C , Schmitt I , Scheidegger C . 2012 Genetic basis of self‐incompatibility in the lichen‐forming fungus *Lobaria pulmonaria* and skewed frequency distribution of mating‐type idiomorphs: implications for conservation. PLoS ONE 7: e51402. 23236495 10.1371/journal.pone.0051402 PMC3517546

[nph14714-bib-0064] Singh G , Dal Grande F , Werth S , Scheidegger C . 2015 Long term consequences of disturbances on reproductive strategies of the rare epiphytic lichen *Lobaria pulmonaria*: clonality a gift and a curse. FEMS Microbiology Ecology 91: 1–11. 10.1093/femsec/fiu009 25764533

[nph14714-bib-0065] Škaloud P , Friedl T , Hallmann C , Beck A , Dal Grande F . 2016 Taxonomic revision and species delimitation of coccoid green algae currently assigned to the genus *Dictyochloropsis* (Trebouxiophyceae, Chlorophyta). Journal of Phycology 52: 599–617. 27135898 10.1111/jpy.12422

[nph14714-bib-0066] Smith CW, Aptroot A, Coppins B, Fletcher A, Gilbert OL, James PW, Wolseley PA, eds. 2009 The lichens of Great Britain and Ireland. London, UK: British Lichen Society.

[nph14714-bib-0067] Snyder RE , Chesson P . 2003 Local dispersal can facilitate coexistence in the presence of permanent spatial heterogeneity. Ecology Letters 6: 301–309.

[nph14714-bib-0068] Soons MB , Ozinga WA . 2005 How important is long‐distance seed dispersal for the regional survival of plant species? Diversity and Distributions 11: 165–172.

[nph14714-bib-0069] Steinberg G . 2015 Cell biology of *Zymoseptoria tritici*: pathogen cell organization and wheat infection. Fungal Genetics and Biology 79: 17–23. 26092785 10.1016/j.fgb.2015.04.002 PMC4502449

[nph14714-bib-0070] Tackenberg O . 2003 Modeling long‐distance dispersal of plant diaspores by wind. Ecological Monographs 73: 173–189.

[nph14714-bib-0071] Tapper R . 1976 Dispersal and changes in local distributions of *Evernia prunastri* and *Ramalina farinacea* . New Phytologist 77: 725–734.

[nph14714-bib-0072] Taschen E , Rousset F , Sauve M , Benoit L , Dubois MP , Richard F , Selosse MA . 2016 How the truffle got its mate: insights from genetic structure in spontaneous and planted Mediterranean populations of *Tuber melanosporum* . Molecular Ecology 25: 5611–5627. 27717090 10.1111/mec.13864

[nph14714-bib-0073] Thomas CD . 2000 Dispersal and extinction in fragmented landscapes. Proceedings of the Royal Society of London B: Biological Sciences 267: 139–145. 10.1098/rspb.2000.0978 PMC1690516 10687818

[nph14714-bib-0074] Vallejo‐Marìn M , Dorken ME , Barrett SCH . 2010 The ecological and evolutionary consequences of clonality for plant mating. Annual Review of Ecology Evolution, and Systematics 41: 193–213.

[nph14714-bib-0075] Vobis G . 1977 Studies on the germination of lichen conidia. The Lichenologist 9: 131–136.

[nph14714-bib-0076] Wagner HH , Werth S , Kalwij JM , Bolli JC , Scheidegger C . 2006 Modelling forest recolonization by an epiphytic lichen using a landscape genetic approach. Landscape Ecology 21: 849–865.

[nph14714-bib-0078] Walser JC , Sperisen C , Soliva M , Scheidegger C . 2003 Fungus‐specific microsatellite primers of lichens: application for the assessment of genetic variation on different spatial scales in *Lobaria pulmonaria* . Fungal Genetics and Biology 40: 72–82. 12948515 10.1016/s1087-1845(03)00080-x

[nph14714-bib-0080] Werth S , Cheenacharoen S , Scheidegger C . 2014 Propagule size is not a good predictor for regional population subdivision or fine‐scale spatial structure in lichenized fungi. Fungal Biology 118: 126–138. 24528636 10.1016/j.funbio.2013.10.009

[nph14714-bib-0081] Werth S , Cornejo C , Scheidegger C . 2013 Characterization of microsatellite loci in the lichen fungus *Lobaria pulmonaria* (Lobariaceae). Applications in Plant Sciences 1: apps. 1200290. 10.3732/apps.1200290 PMC4105370 25202516

[nph14714-bib-0082] Werth S , Scheidegger C . 2012 Congruent genetic structure in the lichen‐forming fungus *Lobaria pulmonaria* and its green‐algal photobiont. Molecular Plant–Microbe Interactions 25: 220–230. 22046957 10.1094/MPMI-03-11-0081

[nph14714-bib-0083] Werth S , Wagner HH , Gugerli F , Holderegger R , Csencsics D , Kalwij JM , Scheidegger C . 2006a Quantifying dispersal and establishment limitation in a population of an epiphytic lichen. Ecology 87: 2037–2046. 16937643 10.1890/0012-9658(2006)87[2037:qdaeli]2.0.co;2

[nph14714-bib-0084] Werth S , Wagner HH , Holderegger R , Kalwij JM , Scheidegger C . 2006b Effect of disturbances on the genetic diversity of an old‐forest associated lichen. Molecular Ecology 15: 911–921. 16599956 10.1111/j.1365-294X.2006.02838.x

[nph14714-bib-0085] Williams CF . 1994 Genetic consequences of seed dispersal in three sympatric forest herbs. II. Microspatial genetic structure within populations. Evolution 48: 1959–1972. 28565156 10.1111/j.1558-5646.1994.tb02226.x

[nph14714-bib-0086] Zhang Y , Zhang D . 2007 Asexual and sexual reproductive strategies in clonal plants. Frontiers of Biology in China 2: 256–262.

[nph14714-bib-0087] Zoller S , Lutzoni F , Scheidegger C . 1999 Genetic variation within and among populations of the threatened lichen *Lobaria pulmonaria* in Switzerland and implications for its conservation. Molecular Ecology 8: 2049–2059. 10632856 10.1046/j.1365-294x.1999.00820.x

