# Supplementary material for: Discovery of long‐distance gamete dispersal in a lichen‐forming ascomycete
Source: New Phytol. 2017 Aug 7;216(1):216–26. doi: 10.1111/nph.14714 (PMC5655791; doi:10.1111/nph.14714)

***New Phytologist* Supporting Information**

Article title: **DISCOVERY OF LONG-DISTANCE GAMETE DISPERSAL IN A LICHEN-FORMING ASCOMYCETE**

Authors: Cecilia Ronnås, Silke Werth, Otso Ovaskainen, Gergely Várkonyi, Christoph Scheidegger and Tord Snäll

Article acceptance date: 16 June 2017

The following Supporting Information is available for this article:

Robustness of results

The results were robust and did not depend on the modelling criteria used; with all methods, we found long-distance dispersal of gametes and ascospores and short-distance dispersal of clonal propagules (Supporting Information 1–5).

Fig. S1 Effective dispersal distance and probability of deposition to at least distance x for clonal propagules, gametes, and ascospores in the epiphytic lichen-forming ascomycete *Lobaria pulmonaria*. Panels **A**–**C** show the probability density function (PDF ; $\times 10^3$) for deposition, i.e. the probability that a dispersal unit deposits between any two distances values. This probability is given by the integral of the PDF between those two values. Panels **D**–**F** show the dispersal functions, i.e. the probability that the dispersal unit deposits at a location at least at distance x from its source. Black lines show the posterior medians, and the shading shows the upper and lower 25 % (dark green) and 2.5% (light green) posterior quantiles, respectively. Note the different scaling of the x-axes. The figure represents analysis I, clones dropped (see Materials and methods)

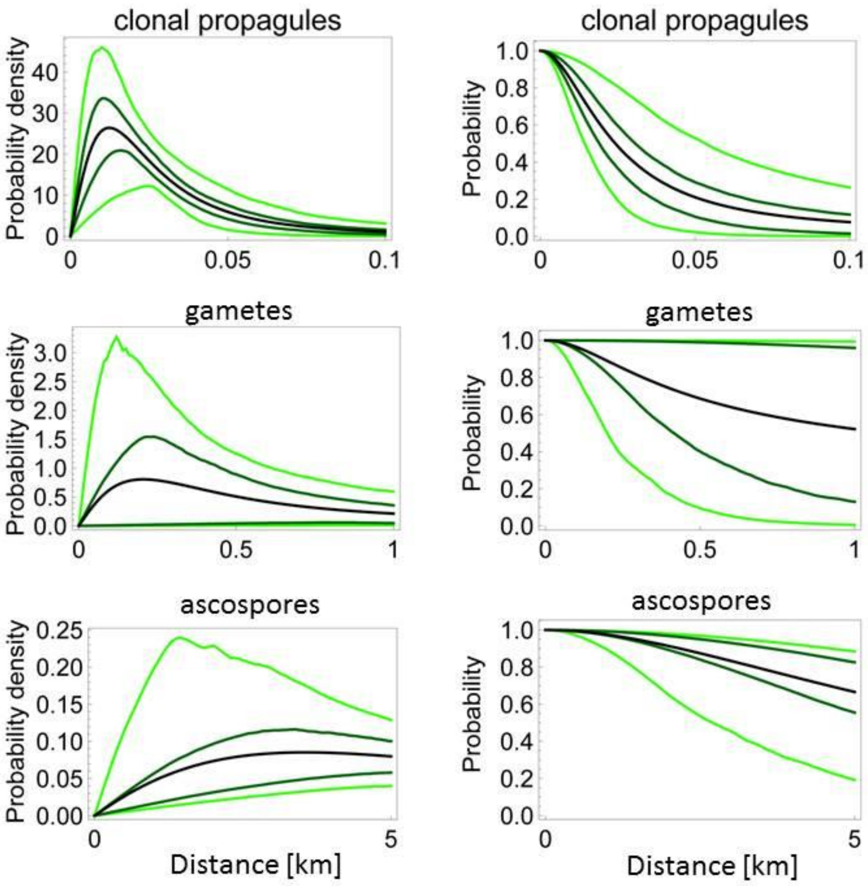

Fig. S2 Effective dispersal distance and probability of deposition to at least distance x for clonal propagules, gametes, and ascospores in the epiphytic lichen-forming ascomycete *Lobaria pulmonaria* based on analysis II, clones dropped. For further information, see Fig. S1.

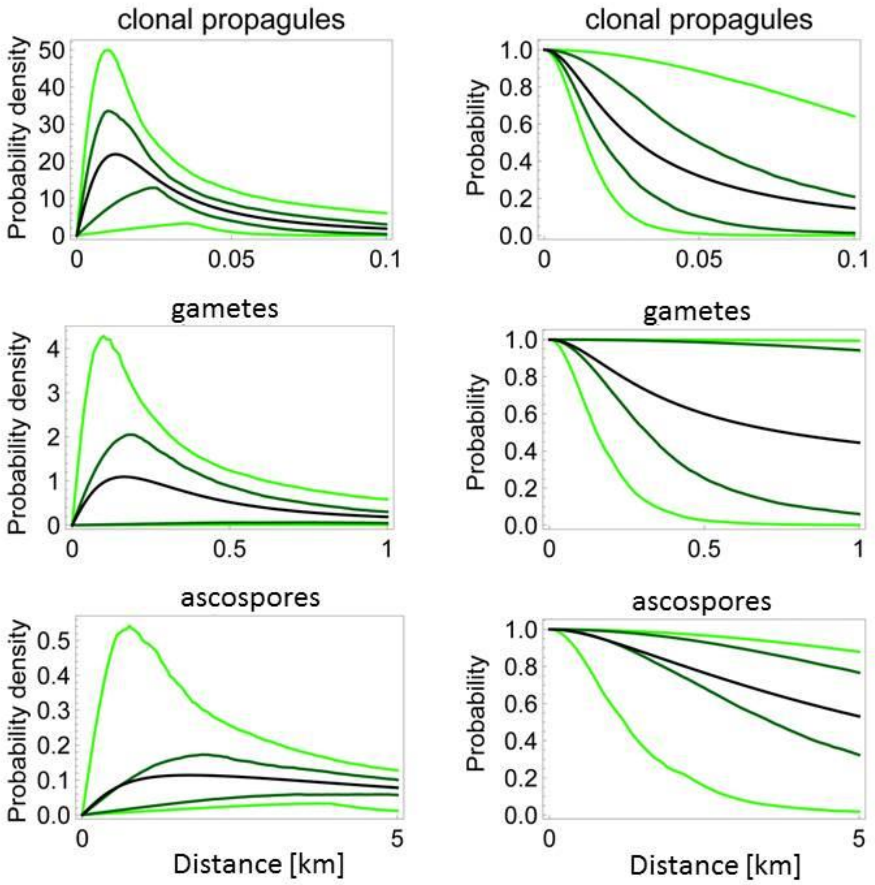

Fig. S3 Effective dispersal distance and probability of deposition to at least distance x for clonal propagules, gametes, and ascospores in the epiphytic lichen-forming ascomycete *Lobaria pulmonaria* based on analysis II, clones not dropped. For further information, see Fig. S1.

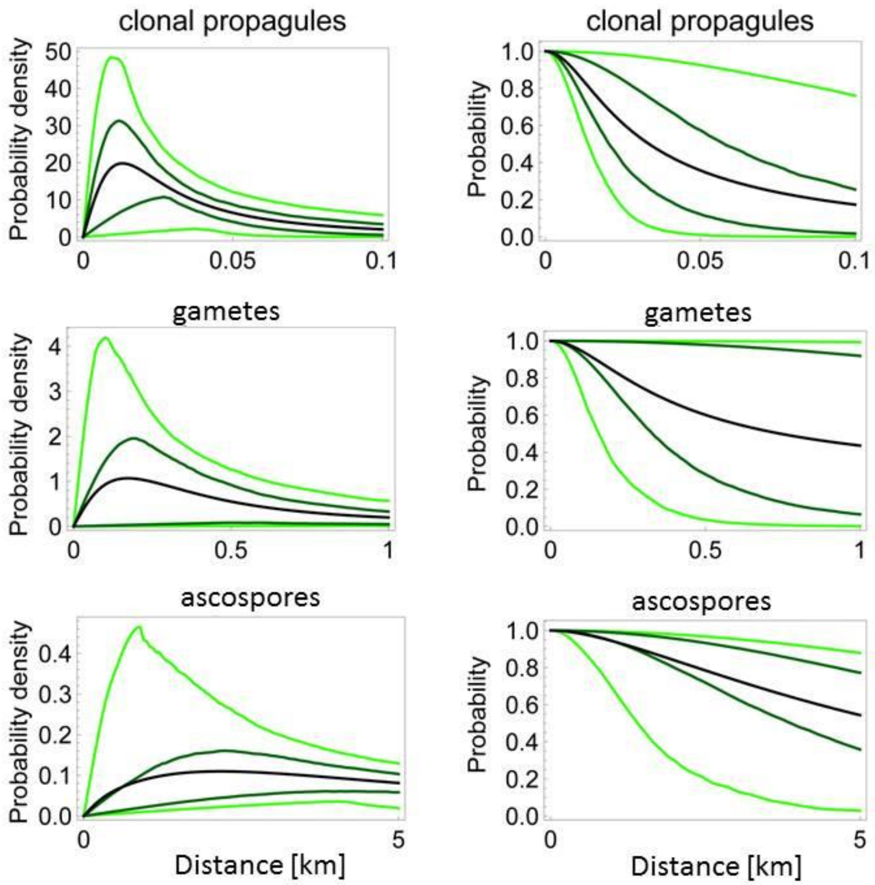

Fig. S4 Effective dispersal distance and probability of deposition to at least distance x for clonal propagules, gametes, and ascospores in the epiphytic lichen-forming ascomycete *Lobaria pulmonaria* based on analysis III, clones dropped. For further information, see Fig. S1.

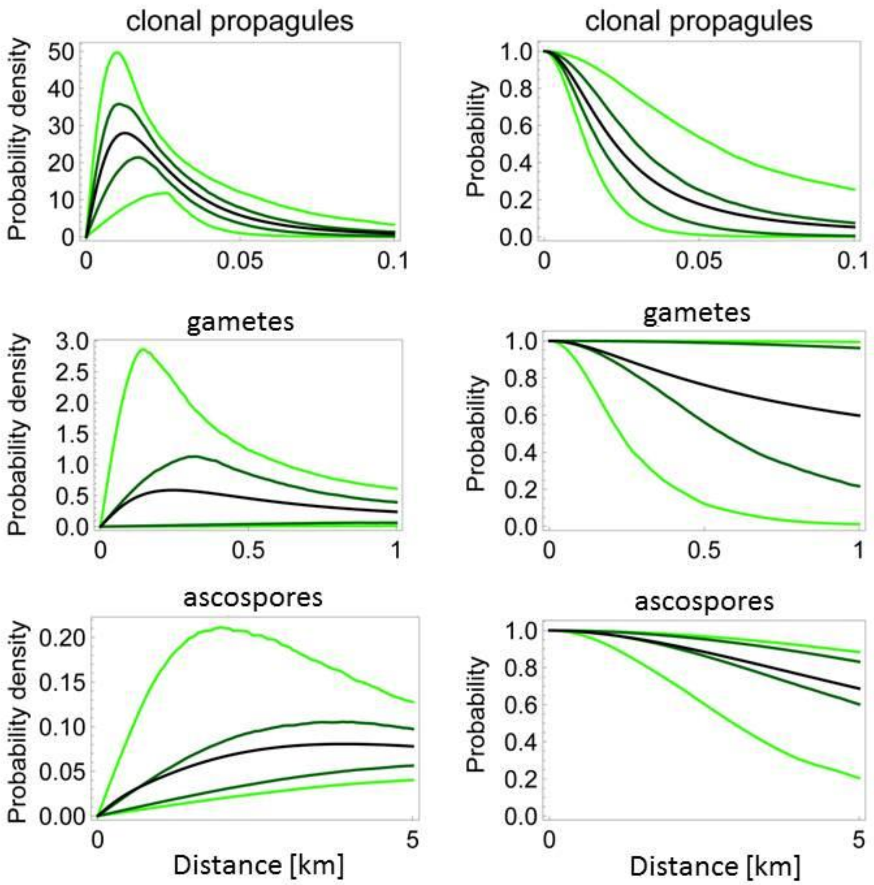

Fig. S5 Effective dispersal distance and probability of deposition to at least distance x for clonal propagules, gametes, and ascospores in the epiphytic lichen-forming ascomycete *Lobaria pulmonaria* based on analysis III, clones not dropped. For further information, see Fig. S1.

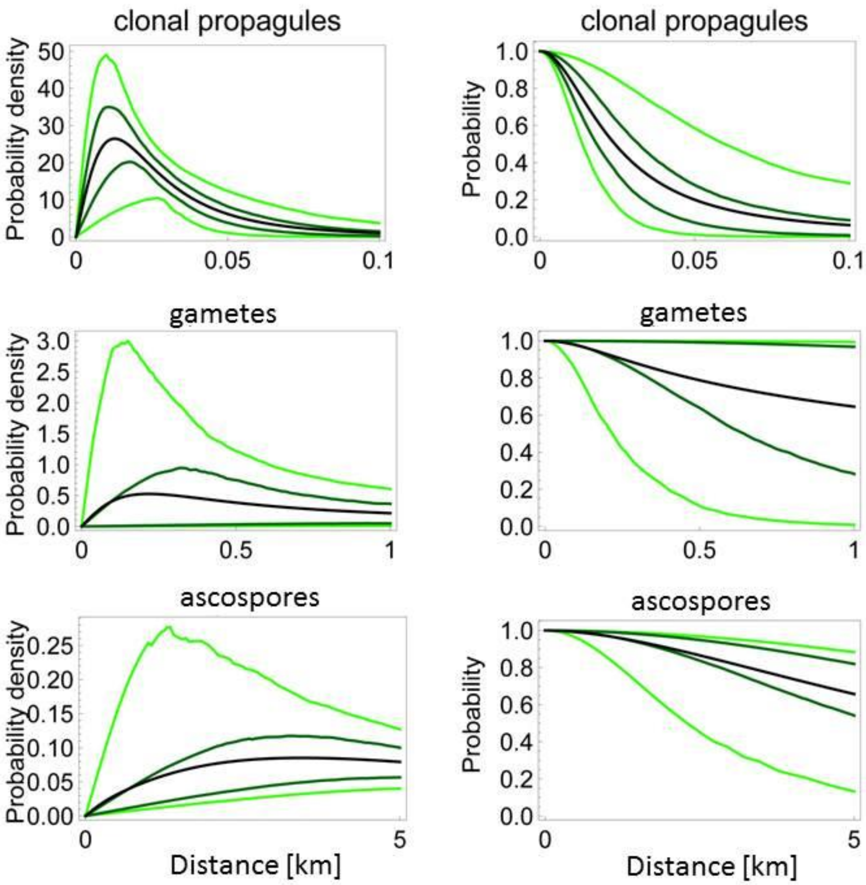

Supplement: Supplementary file 1 — Fig. S1 Effective dispersal distance and probability of deposition to at least distance x for clonal propagules, gametes and ascospores in the epiphytic lichen‐forming ascomycete Lobaria pulmonaria. The figure represents analysis I, clones dropped. Fig. S2 Effective dispersal distance and probability of deposition to at least distance x for clonal propagules, gametes and ascospores in the epiphytic lichen‐forming ascomycete Lobaria pulmonaria based on analysis II, clones dropped. Fig. S3 Effective dispersal distance and probability of deposition to at least distance x for clonal propagules, gametes and ascospores in the epiphytic lichen‐forming ascomycete Lobaria pulmonaria based on analysis II, clones not dropped. Fig. S4 Effective dispersal distance and probability of deposition to at least distance x for clonal propagules, gametes and ascospores in the epiphytic lichen‐forming ascomycete Lobaria pulmonaria based on analysis III, clones dropped. Fig. S5 Effective dispersal distance and probability of deposition to at least distance x for clonal propagules, gametes and ascospores in the epiphytic lichen‐forming ascomycete Lobaria pulmonaria based on analysis III, clones not dropped. [file NPH-216-216-s001.pdf]
